# 3,3-Bis(methyl­sulfan­yl)-1-(4-nitro­phen­yl)prop-2-en-1-one

**DOI:** 10.1107/S1600536813014542

**Published:** 2013-06-08

**Authors:** Guan-Neng Yu, Jian-Hui Xia, Zhao-Hui Xu, Li-Ben Wang, Chu-Yi Yu

**Affiliations:** a College of Chemistry and Chemical Engineering, Jiang Xi Normal University, Nanchang, Jiang Xi 330022, People’s Republic of China; b Beijing National Laboratory for Molecular Science (BNLMS), CAS Key Laboratory of Molecular Recognition and Function, Institute of Chemistry, Chinese Academy of Sciences, Beijing 100190, People’s Republic of China

## Abstract

In the title compound, C_11_H_11_NO_3_S_2_, the S—C*sp* ^2^ bonds are shorter [1.746 (3) and 1.750 (2) Å] than the S—CH_3_ bonds [1.794 (3) and 1.806 (3) Å], which we attribute to *d*–π inter­actions between the S atoms and the C=C bond. The 1,1-bis­(methyl­sulfan­yl)-3-oxo­propyl­ene fragment and the 4-nitro­phenyl group are both almost planar, with the largest deviations from their mean planes being 0.053 (1) and 0.017 (2) Å, respectively. The dihedral angle between the two planes is 35.07 (7)°. Mol­ecules in the crystal are linked into a three-dimensional network by C—H⋯S and C—H⋯O hydrogen bonds.

## Related literature

For the synthesis of the title compound, see: Huang & Liu (1989 ▶). For applications, see: Barun *et al.* (2000 ▶); Kuettel *et al.* (2007 ▶). For general background on ketene aminals, see: Huang & Wang (1994 ▶).
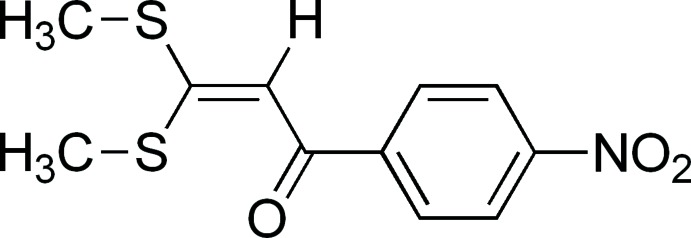


## Experimental

### 

#### Crystal data

C_11_H_11_NO_3_S_2_
*M* *_r_* = 269.33
Triclinic, 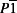

*a* = 7.917 (2) Å
*b* = 8.739 (2) Å
*c* = 9.574 (3) Å
α = 70.415 (13)°
β = 81.985 (14)°
γ = 73.283 (13)°
*V* = 597.0 (3) Å^3^
*Z* = 2
Mo *K*α radiation
μ = 0.44 mm^−1^
*T* = 173 K
0.27 × 0.24 × 0.05 mm

#### Data collection

Rigaku Saturn724+ CCD diffractometer
Absorption correction: multi-scan (*CrystalClear*; Rigaku, 2007 ▶) *T*_min_ = 0.581, *T*_max_ = 1.000
7843 measured reflections
2719 independent reflections
2394 reflections with *I* > 2σ(*I*)
*R*_int_ = 0.053

#### Refinement

*R*[*F* ^2^ > 2σ(*F* ^2^)] = 0.055
*wR*(*F* ^2^) = 0.115
*S* = 1.15
2719 reflections
156 parameters
H-atom parameters constrained
Δρ_max_ = 0.29 e Å^−3^
Δρ_min_ = −0.23 e Å^−3^

### 

Data collection: *CrystalClear* (Rigaku, 2007 ▶); cell refinement: *CrystalClear*; data reduction: *CrystalClear*; program(s) used to solve structure: *SHELXS97* (Sheldrick, 2008 ▶); program(s) used to refine structure: *SHELXL97* (Sheldrick, 2008 ▶); molecular graphics: *OLEX2* (Dolomanov *et al.*, 2009 ▶); software used to prepare material for publication: *SHELXL97*.

## Supplementary Material

Crystal structure: contains datablock(s) I, global. DOI: 10.1107/S1600536813014542/fy2092sup1.cif

Structure factors: contains datablock(s) I. DOI: 10.1107/S1600536813014542/fy2092Isup2.hkl

Supplementary material file. DOI: 10.1107/S1600536813014542/fy2092Isup3.cml

Additional supplementary materials:  crystallographic information; 3D view; checkCIF report

**Fig. 1.**
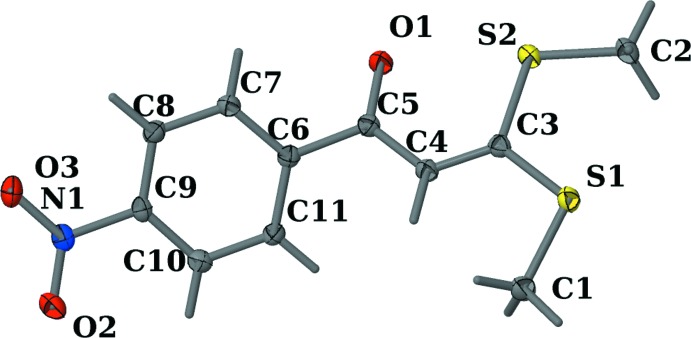
The molecular structure of (I). Displacement ellipsoids are drawn at the 30% probability level.

Enhanced figure: interactive version of Fig. 1

## Figures and Tables

**Table 1:** Hydrogen-bond geometry (Å, °)

*D*—H⋯*A*	*D*—H	H⋯*A*	*D*⋯*A*	*D*—H⋯*A*
C11—H11⋯S2^i^	0.95	2.93	3.614 (3)	130
C8—H8⋯O3^ii^	0.95	2.63	3.204 (3)	119
C2—H2*B*⋯O3^iii^	0.98	2.68	3.297 (4)	122
C10—H10⋯O1^iv^	0.95	2.66	3.602 (3)	171
C1—H1*C*⋯O1^v^	0.98	2.59	3.551 (3)	167
C7—H7⋯O2^vi^	0.95	2.55	3.499 (3)	179

Symmetry codes: (i) 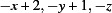; (ii) 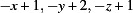; (iii) 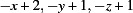; (iv) 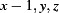; (v) 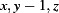; (vi) 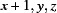.

## supplementary crystallographic information

### Comment

Heterocyclic ketene aminals are important and versatile starting materials for the synthesis of a wide variety of fused heterocycles (Huang & Wang, 1994). In this paper, we report the crystal structure of the title compound, which is a precursor of heterocyclic ketene aminals. The shortening of bonds C3—S1 [1.746 (3) Å] and C3—S2 [1.750 (2) Å] with respect to bonds C1—S1 [1.794 (3) Å] and C2—S2 [1.806 (3) Å] is attributed to *d*-π interactions between sulfur and the C=C bond. The dihedral angle between 4-nitrophenyl group and (C1, C2, S1, S2, C3, C4, C5, O1) is 35.07 (7)°. In the structure of the title compound, molecules are connected through intermolecular C—H···S and C—H···O hydrogen bonding (Table 1) into a three-dimensional network.

### Experimental

The title compound was prepared according to the method of Huang & Liu (1989). m.p. 435–437 K. MS: m/*z* = 269 (*M*^+^). IR: 1615 (C=O), 1590 (C=C), 1512, 1345 (NO_2_) cm^-1. 1^H-NMR: *δ* = 8.33 (d, 2H), 8.07 (d, 2H), 6.73 (s, lH), 2.60 (s, 3H), 2.57 (s, 3H) p.p.m.. *^l^*^3^C-NMR: *δ* =183.5, 170.6, 149.9, 144.9, 129.0, 124.1, 109.0, 17.9, 15.6 p.p.m.. Anal. Calc. for C_11_H_11_N0_3_S_2_: C, 49.05; H, 4.12; N, 5.20. Found C, 49.29; H, 4.23; N, 5.36. Single crystals of the title compound suitable for X-ray diffraction analysis were obtained from ethanol solution by slow evaporation after a week.

### Refinement

H atoms were positioned geometrically and refined using a riding model, with C—H = 0.98 (C1, C3) or 0.95 Å (C6) and with *U*_iso_(H) = 1.5 times *U*_eq_(C) (methyl groups) or with *U*_iso_(H) = 1.2 times *U*_eq_(C) (benzene ring).

### Crystal data

**Table:**

C_11_H_11_NO_3_S_2_	*Z* = 2
*M**_r_* = 269.33	*F*(000) = 280
Triclinic, *P*1	*D*_x_ = 1.498 Mg m^−^^3^
*a* = 7.917 (2) Å	Mo *K*α radiation, λ = 0.71073 Å
*b* = 8.739 (2) Å	Cell parameters from 2392 reflections
*c* = 9.574 (3) Å	θ = 2.3–32.6°
α = 70.415 (13)°	µ = 0.44 mm^−^^1^
β = 81.985 (14)°	*T* = 173 K
γ = 73.283 (13)°	Plate, yellow
*V* = 597.0 (3) Å^3^	0.27 × 0.24 × 0.05 mm

### Data collection

**Table:**

Saturn724+ CCD diffractometer	2719 independent reflections
Radiation source: sealed tube	2394 reflections with *I* > 2σ(*I*)
Graphite monochromator	*R*_int_ = 0.053
ω scans at fixed χ = 45°	θ_max_ = 27.5°, θ_min_ = 2.3°
Absorption correction: multi-scan (*CrystalClear*; Rigaku, 2007)	*h* = −10→10
*T*_min_ = 0.581, *T*_max_ = 1.000	*k* = −11→11
7843 measured reflections	*l* = −12→12

### Refinement

**Table:**

Refinement on *F*^2^	Primary atom site location: structure-invariant direct methods
Least-squares matrix: full	Secondary atom site location: difference Fourier map
*R*[*F*^2^ > 2σ(*F*^2^)] = 0.055	Hydrogen site location: inferred from neighbouring sites
*wR*(*F*^2^) = 0.115	H-atom parameters constrained
*S* = 1.15	*w* = 1/[σ^2^(*F*_o_^2^) + (0.0306*P*)^2^ + 0.3933*P*] where *P* = (*F*_o_^2^ + 2*F*_c_^2^)/3
2719 reflections	(Δ/σ)_max_ < 0.001
156 parameters	Δρ_max_ = 0.29 e Å^−^^3^
0 restraints	Δρ_min_ = −0.23 e Å^−^^3^

### Special details

**Table:**

Geometry. All e.s.d.'s (except the e.s.d. in the dihedral angle between two l.s. planes) are estimated using the full covariance matrix. The cell e.s.d.'s are taken into account individually in the estimation of e.s.d.'s in distances, angles and torsion angles; correlations between e.s.d.'s in cell parameters are only used when they are defined by crystal symmetry. An approximate (isotropic) treatment of cell e.s.d.'s is used for estimating e.s.d.'s involving l.s. planes.
Refinement. Refinement of *F*^2^ against ALL reflections. The weighted *R*-factor *wR* and goodness of fit *S* are based on *F*^2^, conventional *R*-factors *R* are based on *F*, with *F* set to zero for negative *F*^2^. The threshold expression of *F*^2^ > σ(*F*^2^) is used only for calculating *R*-factors(gt) *etc*. and is not relevant to the choice of reflections for refinement. *R*-factors based on *F*^2^ are statistically about twice as large as those based on *F*, and *R*- factors based on ALL data will be even larger.

### Fractional atomic coordinates and isotropic or equivalent isotropic displacement parameters (Å^2^)

**Table:**

	*x*	*y*	*z*	*U*_iso_*/*U*_eq_	
S1	1.24009 (8)	0.08728 (8)	0.14324 (8)	0.03498 (19)	
S2	1.38394 (8)	0.37041 (8)	0.12260 (7)	0.03303 (18)	
O1	1.1193 (2)	0.6171 (2)	0.1933 (2)	0.0386 (4)	
O2	0.2086 (2)	0.7642 (3)	0.4617 (2)	0.0429 (5)	
O3	0.3377 (3)	0.8672 (3)	0.5789 (2)	0.0518 (6)	
N1	0.3402 (3)	0.7914 (3)	0.4916 (2)	0.0338 (5)	
C1	1.0268 (3)	0.0451 (3)	0.1766 (3)	0.0404 (6)	
H1A	0.9467	0.1298	0.1022	0.061*	
H1B	0.9799	0.0492	0.2759	0.061*	
H1C	1.0375	−0.0668	0.1698	0.061*	
C2	1.5600 (3)	0.2069 (4)	0.0782 (3)	0.0398 (6)	
H2A	1.5239	0.1753	0.0001	0.060*	
H2B	1.5857	0.1086	0.1670	0.060*	
H2C	1.6660	0.2481	0.0434	0.060*	
C3	1.2001 (3)	0.2871 (3)	0.1613 (3)	0.0280 (5)	
C4	1.0404 (3)	0.3722 (3)	0.2043 (3)	0.0297 (5)	
H4	0.9447	0.3218	0.2225	0.036*	
C5	1.0078 (3)	0.5353 (3)	0.2239 (3)	0.0299 (5)	
C6	0.8303 (3)	0.6031 (3)	0.2912 (3)	0.0273 (5)	
C7	0.8213 (3)	0.6942 (3)	0.3879 (3)	0.0309 (5)	
H7	0.9257	0.7142	0.4083	0.037*	
C8	0.6607 (3)	0.7558 (3)	0.4548 (3)	0.0321 (5)	
H8	0.6540	0.8155	0.5229	0.038*	
C9	0.5109 (3)	0.7280 (3)	0.4199 (3)	0.0280 (5)	
C10	0.5143 (3)	0.6403 (3)	0.3229 (3)	0.0300 (5)	
H10	0.4088	0.6243	0.3002	0.036*	
C11	0.6762 (3)	0.5765 (3)	0.2597 (3)	0.0292 (5)	
H11	0.6823	0.5139	0.1941	0.035*	

### Atomic displacement parameters (Å^2^)

**Table:**

	*U*^11^	*U*^22^	*U*^33^	*U*^12^	*U*^13^	*U*^23^
S1	0.0283 (3)	0.0309 (3)	0.0462 (4)	−0.0060 (3)	0.0047 (3)	−0.0165 (3)
S2	0.0256 (3)	0.0382 (4)	0.0383 (4)	−0.0103 (3)	0.0037 (3)	−0.0160 (3)
O1	0.0320 (10)	0.0386 (10)	0.0522 (12)	−0.0155 (8)	0.0104 (8)	−0.0227 (9)
O2	0.0277 (10)	0.0525 (12)	0.0472 (12)	−0.0118 (9)	0.0037 (8)	−0.0150 (10)
O3	0.0423 (12)	0.0650 (14)	0.0573 (13)	−0.0078 (10)	0.0109 (10)	−0.0411 (12)
N1	0.0305 (11)	0.0329 (11)	0.0342 (12)	−0.0064 (9)	0.0031 (9)	−0.0090 (10)
C1	0.0329 (14)	0.0321 (13)	0.0599 (18)	−0.0101 (12)	0.0026 (13)	−0.0194 (13)
C2	0.0242 (13)	0.0458 (16)	0.0479 (16)	−0.0069 (12)	0.0054 (11)	−0.0174 (13)
C3	0.0272 (12)	0.0304 (12)	0.0264 (12)	−0.0082 (10)	−0.0004 (9)	−0.0088 (10)
C4	0.0274 (12)	0.0296 (12)	0.0332 (13)	−0.0093 (10)	0.0022 (10)	−0.0108 (11)
C5	0.0295 (12)	0.0326 (13)	0.0303 (13)	−0.0114 (11)	0.0019 (10)	−0.0118 (10)
C6	0.0293 (12)	0.0252 (11)	0.0271 (12)	−0.0077 (10)	0.0005 (9)	−0.0077 (10)
C7	0.0270 (12)	0.0320 (13)	0.0371 (14)	−0.0109 (10)	0.0023 (10)	−0.0139 (11)
C8	0.0357 (14)	0.0327 (13)	0.0323 (13)	−0.0111 (11)	0.0010 (10)	−0.0150 (11)
C9	0.0271 (12)	0.0268 (12)	0.0265 (12)	−0.0052 (10)	0.0027 (9)	−0.0067 (10)
C10	0.0275 (12)	0.0292 (12)	0.0326 (13)	−0.0086 (10)	−0.0031 (10)	−0.0070 (10)
C11	0.0304 (12)	0.0306 (12)	0.0295 (12)	−0.0073 (10)	−0.0016 (10)	−0.0136 (10)

### Geometric parameters (Å, º)

**Table:**

S1—C3	1.746 (3)	C3—C4	1.357 (3)
S1—C1	1.794 (3)	C4—C5	1.444 (3)
S2—C3	1.750 (2)	C4—H4	0.9500
S2—C2	1.806 (3)	C5—C6	1.505 (3)
O1—C5	1.236 (3)	C6—C7	1.392 (3)
O2—N1	1.223 (3)	C6—C11	1.395 (3)
O3—N1	1.223 (3)	C7—C8	1.389 (3)
N1—C9	1.473 (3)	C7—H7	0.9500
C1—H1A	0.9800	C8—C9	1.381 (3)
C1—H1B	0.9800	C8—H8	0.9500
C1—H1C	0.9800	C9—C10	1.381 (3)
C2—H2A	0.9800	C10—C11	1.385 (3)
C2—H2B	0.9800	C10—H10	0.9500
C2—H2C	0.9800	C11—H11	0.9500
			
C3—S1—C1	104.01 (12)	C5—C4—H4	118.2
C3—S2—C2	103.83 (12)	O1—C5—C4	123.4 (2)
O2—N1—O3	123.4 (2)	O1—C5—C6	119.3 (2)
O2—N1—C9	118.3 (2)	C4—C5—C6	117.2 (2)
O3—N1—C9	118.3 (2)	C7—C6—C11	119.6 (2)
S1—C1—H1A	109.5	C7—C6—C5	118.6 (2)
S1—C1—H1B	109.5	C11—C6—C5	121.8 (2)
H1A—C1—H1B	109.5	C8—C7—C6	120.4 (2)
S1—C1—H1C	109.5	C8—C7—H7	119.8
H1A—C1—H1C	109.5	C6—C7—H7	119.8
H1B—C1—H1C	109.5	C9—C8—C7	118.3 (2)
S2—C2—H2A	109.5	C9—C8—H8	120.9
S2—C2—H2B	109.5	C7—C8—H8	120.9
H2A—C2—H2B	109.5	C10—C9—C8	123.0 (2)
S2—C2—H2C	109.5	C10—C9—N1	118.4 (2)
H2A—C2—H2C	109.5	C8—C9—N1	118.6 (2)
H2B—C2—H2C	109.5	C9—C10—C11	118.0 (2)
C4—C3—S1	123.37 (19)	C9—C10—H10	121.0
C4—C3—S2	121.71 (19)	C11—C10—H10	121.0
S1—C3—S2	114.92 (14)	C10—C11—C6	120.8 (2)
C3—C4—C5	123.6 (2)	C10—C11—H11	119.6
C3—C4—H4	118.2	C6—C11—H11	119.6
			
C1—S1—C3—C4	5.0 (3)	C5—C6—C7—C8	−178.6 (2)
C1—S1—C3—S2	−176.01 (14)	C6—C7—C8—C9	−1.6 (4)
C2—S2—C3—C4	178.0 (2)	C7—C8—C9—C10	0.7 (4)
C2—S2—C3—S1	−0.99 (17)	C7—C8—C9—N1	179.4 (2)
S1—C3—C4—C5	178.02 (19)	O2—N1—C9—C10	−0.3 (3)
S2—C3—C4—C5	−0.9 (4)	O3—N1—C9—C10	179.1 (2)
C3—C4—C5—O1	5.3 (4)	O2—N1—C9—C8	−179.0 (2)
C3—C4—C5—C6	−172.2 (2)	O3—N1—C9—C8	0.4 (3)
O1—C5—C6—C7	−34.5 (3)	C8—C9—C10—C11	0.7 (4)
C4—C5—C6—C7	143.0 (2)	N1—C9—C10—C11	−178.0 (2)
O1—C5—C6—C11	145.8 (2)	C9—C10—C11—C6	−1.2 (4)
C4—C5—C6—C11	−36.7 (3)	C7—C6—C11—C10	0.4 (4)
C11—C6—C7—C8	1.1 (4)	C5—C6—C11—C10	−180.0 (2)

### Hydrogen-bond geometry (Å, º)

**Table:**

*D*—H···*A*	*D*—H	H···*A*	*D*···*A*	*D*—H···*A*
C11—H11···S2^i^	0.95	2.93	3.614 (3)	130
C8—H8···O3^ii^	0.95	2.63	3.204 (3)	119
C2—H2*B*···O3^iii^	0.98	2.68	3.297 (4)	122
C10—H10···O1^iv^	0.95	2.66	3.602 (3)	171
C1—H1*C*···O1^v^	0.98	2.59	3.551 (3)	167
C7—H7···O2^vi^	0.95	2.55	3.499 (3)	179

Symmetry codes: (i) −*x*+2, −*y*+1, −*z*; (ii) −*x*+1, −*y*+2, −*z*+1; (iii) −*x*+2, −*y*+1, −*z*+1; (iv) *x*−1, *y*, *z*; (v) *x*, *y*−1, *z*; (vi) *x*+1, *y*, *z*.
